# Deoxygenation Trends and Their Multivariate Association with Self-Reported Fatigue in Post-COVID Syndrome

**DOI:** 10.3390/biomedicines13061371

**Published:** 2025-06-03

**Authors:** Anja-Maria Ladek, Marianna Lucio, Andreas Weiß, Thomas Knauer, Helena Sarmiento, Miriam Ilgner, Marie Jakobi, Laura Barteczko, Marion Ganslmayer, Jürgen Rech, Antonio Bergua, Christian Y. Mardin, Bettina Hohberger

**Affiliations:** 1 Department of Ophthalmology, Universitätsklinikum Erlangen, Friedrich-Alexander-Universität Erlangen-Nürnberg, 91054 Erlangen, Germany; 2 PhD Medical Sciences, Department of Human Medicine, Paracelsus Medical University, 5020 Salzburg, Austria; 3 Research Unit Analytical BioGeoChemistry, Helmholtz Zentrum München, 85764 Neuherberg, Germany; 4 Rotkreuzklinik Lindenberg, 88161 Lindenberg, Germany; 5 Department of Internal Medicine 1, Universitätsklinikum Erlangen, Friedrich-Alexander-Universität Erlangen-Nürnberg, 91054 Erlangen, Germany; 6 Department of Internal Medicine 3—Rheumatology and Immunology, Deutsches Zentrum Immuntherapie and Center for Rare Diseases Erlangen, Universitätsklinikum Erlangen, Friedrich-Alexander-Universität Erlangen-Nürnberg, 91054 Erlangen, Germany

**Keywords:** Post-COVID syndrome, NIRS, oxygenation, fatigue, PEM, deoxygenation

## Abstract

**Background/Objectives:** A relevant subgroup of post-COVID-19 syndrome (PCS) patients suffers from post-exertional malaise (PEM) and cardiovascular or neurological symptoms, impairing daily functioning up to becoming even house- or bedbound. Recent data suggest that PCS summarizes different subgroups, one of them being characterized by an impaired microcirculation. Thus, the aim of the present study was to investigate local deoxygenation, measured with non-invasive near-infrared regional spectroscopy (NIRS), and its association with self-reported fatigue in patients with PCS compared to controls in light exercise. **Methods:** 150 participants (100 PCS patients and 50 controls) were recruited. PEM was assessed using FACIT, Chalder, and Bell scoring and Canadian Criteria. NIRS was used to measure local oxygenation while kneading a stress ball and during recovery. **Results:** PCS patients showed fatigue scores of 30 (Bell score), 20.6 (FACIT fatigue score), and 9.914 (Chalder fatigue score). Decreased deoxygenation peaks at the start of exercise were observed in patients with PCS, compared to controls (*p* = 0.0002). Multivariate analysis identified a subgroup, showing an association between strong fatigue and restricted oxygenation dynamics. **Conclusions:** NIRS could be a potential tool to assess deoxygenation deficits even in moderate to severely impaired PCS patients using light exercise protocols.

## 1. Introduction

Approximately 6% of patients with acute COVID-19 have persisting post-acute sequelae for at least 12 weeks after acute infection, thus suffering from long COVID (LC)/post-COVID-19-syndrome (PCS) according to the WHO [1,2]. The prevalence varies depending on cohorts and vaccination status. More than 200 symptoms characterize PCS; the most frequently reported are fatigue, brain fog, post-exertional malaise (PEM), cardiovascular symptoms, and neurological conditions, including cognitive dysfunction and sleep and sensorimotor disturbances [1,3,4,5]. Among PCS patients, those who are classified as medium-severe to severely affected are largely wheelchair and housebound. Their main symptom, PEM, restricts their daily life by experiencing symptoms at rest with exertion-triggered exacerbations of symptoms, often after a time lag. The diversity of PCS symptoms is likely triggered by different molecular mechanisms, arguing for different PCS subtypes [6,7,8,9,10]. It is assumed that immune and autoimmune dysregulation (e.g., functional autoantibodies targeting G-protein coupled receptors) [11], persisting viral reservoirs or viral reactivation [12], abnormal blood clotting [10], and endothelial dysfunctions act independently or interact in the complex pathogenesis of PCS.

A subgroup of patients with PCS shows a restricted capillary microcirculation, linked to chronic fatigue (CF) [13]. It can be assumed that restricted capillary perfusion can be induced or even induce endothelial dysfunction and impaired oxygenation (reviewed in [14]), further limiting perfusion and mitochondrial function (reviewed in [15]). In addition, these patients’ group yielded functional autoantibodies, targeting G-protein coupled receptors (GPCR-fAAb) associated with the restricted microcirculation [16]. Of interest, the neutralization of GPCR-fAAb by BC007 (Berlin Cures GmbH, Berlin, Germany) was accompanied by an improvement in capillary microcirculation and PCS symptoms [17]. The data of a phase IIa study with BC007 in patients with PCS confirmed the improvement of PCS symptoms after the neutralization of GPCR-fAAb [18].

Regional oxygenation and changes in hemoglobin (Hb) or myoglobin (Mb) oxygenation are of interest, considering restricted capillary microcirculation. This can be measured with near-infrared spectroscopy (NIRS), which is available in portable, relatively cost-effective devices and widely used clinically in intensive care and anaesthesiology and research on exercise physiology [19,20]. As only the muscle beneath the sensor is measured, full-body exercise is not required for analysis. Therefore, it could be a safer, readily available option to investigate oxygenation abnormalities in PCS compared to cardiopulmonary exercise testing (reviewed in [21]), avoiding triggering PEM.

The study aimed to investigate muscle (de)oxygenation during light and local exercise, assessed using NIRS, in patients with PCS compared to controls. In addition, a multivariate analysis was performed to explore the relationship between NIRS-derived oxygenation metrics and self-reported fatigue.

## 2. Materials and Methods

### 2.1. Patients

A total of 150 participants were recruited at the Department of Ophthalmology, Universitätsklinikum Erlangen, Friedrich Alexander University of Erlangen-Nürnberg as part of the disCOVer 2.0 study: 100 patients with PCS (55 female, 45 male) and 50 controls (27 female, 23 male). Demographic data, including age, sex, positive PCR report, relevant medical history, and PCS symptom duration, were collected from all patients. Inclusion criteria for patients with PCS were persistent post-acute sequelae of COVID-19 for at least 3 months after a polymerase chain reaction (PCR)-verified COVID-19 infection. On the day of the investigation, participants were asked to self-report on four different questionnaires targeting fatigue. Fulfillment of the Canadian Criteria for Myalgic Encephalomyelitis/Chronic Fatigue Syndrome (ME/CFS), a questionnaire facilitating the diagnostics of ME/CFS according to the international consensus criteria [22]. The Chalder fatigue scale, an 11-item questionnaire assessing physical and psychological fatigue with high reliability in ME/CFS studies and occupational research, a high score expresses high fatigue [23]. The Bell score, which has also been developed to judge the severity of chronic fatigue syndrome, the activity that can be performed, and the frequency of symptoms [24]. The Bell score is counter-proportional to fatigue, with healthy people scoring a maximum of 100. Lastly, the FACIT fatigue score asks the patient to judge how applicable certain statements are regarding their fatigue symptoms during the past 7 days. It also targets physical- and psychological fatigue and scores counter proportionally to fatigue [25].

Several steps were taken to reduce known confounders: Baum [26] and colleagues found that the severity of fatigue in most cases did not correlate with cardiovascular diseases or echocardiographic findings, except in patients with significant functional impairments. Additionally, the study by Thiele et al. [27] showed that fatigue was the most common symptom 6 months after COVID-19, but it did not correlate with the variables of lung function tests (LFT) or left ventricular ejection fraction (LVEF). Lung abnormalities as well as cardiological issues have already been excluded in previous studies initiated by the respective general practitioners, as well as pulmonologists and cardiologists. This has ultimately led to these patients being referred to a POST-COVID center for further clarification, as these findings were considered unremarkable. Moreover, these were reviewed by a physician of the study team and those with any pathologic findings (e.g., oxygen diffusion impairment, restrictive or obstructive lung function aberrations or heart valve prolapse or insufficiency, abnormal contractility or abnormal ejection fractions) were excluded. All PCS patients were also seen by a licensed internal medicine physician during the study and their previous medical history was reviewed; those with a potential organic cause of medium to severe fatigue other than PCS due to other immune diseases, tumors or organ diseases like overt primary heart, lung, liver, thyroid or kidney disease were excluded.

Although measures were taken to exclude possible confounders, the origin of fatigue for PCS on an individual basis is unknown and could include many causes, including neurological, autonomic, muscular, cardiovascular, hematologic, or mitochondrial alterations [8,9,15]. This causes the PCS group to be likely heterogeneous in its cause of fatigue, potentially obscuring the result and may account for the high variance. All participants signed a written informed consent form, which included giving permission to submit the anonymized clinical data in a scientific publication, in agreement with the Declaration of Helsinki and with permission from the ethics commission (no: 295-20-B).

### 2.2. NIRS

The Masimo O3^®^ regional oximeter with Delta cHbi (Masimo Corporation, Irvine, CA, USA) was used to measure regional muscle oxygenation of the underarm flexors. The device uses near-infrared spectroscopy (NIRS) based on diffuse continuous wave multidistance reflection spectroscopy. The sensors have one emitter and two detectors (i.e., one shallow detector and one deep detector), which allows for the selective calculation of oxygenation in deep tissues by excluding the effects of the superficial tissue. The parameters are calculated based on the different absorption coefficients on the basis of the Lambert–Beer law. Recorded values are changes from the baseline of oxygenated Hb and Mb (delta O_2_Hbi; scale: 1 µM from −98.0 to 99.0), deoxygenated Hb and Mb (delta HHbi; scale: 1 µM from −98.0 to 99.0) and total change in Hb and Mb (delta cHbi; scale: 1 µM from −98.0 to 99.0) as well as regional oxygenation (rSO_2_; scale: % change).

While the absorption coefficients of Hb and Mb are too similar to be distinguishable in NIRS, it is generally assumed that the change in oxygenation and deoxygenation as well as total change resulting from Hb changes, since Mb is located inside the muscle and does not change concentrations [19,20,28].

Patients were seated at rest for at least 5 min before starting the investigation. The self-adhering sensor was applied over the underarm flexors. The sensor application spot was shaved in those with thick or dark hair. The sensor was additionally secured and shielded from light with a dark bandage. The measurement baseline was established according to the manufacturer’s instructions. Afterward, some time was taken to ensure stable measurements. Participants were then asked to vigorously knead a stress ball. As the curve variance is already expected to be high, and oxygenation changes from the baseline occur delayed in submaximal exercise [20,29], time was counted from the first changes from the baseline. After 60 s of the first oxygenation changes from the baseline, the participant was asked to suddenly stop and remain relaxed. Oxygenation changes continued to be measured for 2 min after the exercise.

The resulting oxygenation curves recorded the rSO_2_ and the changes from the baseline of hHbi, O_2_Hbi, and cHbi during the light exercise and early recovery. From the resulting oxygenation curves, the area under the curve, the maximum increase overall, the maximum decrease overall, and the time elapsed until the maximum increase or decrease were extracted and included in multivariate analysis (see below).

### 2.3. Statistical Analysis

In the dataset, the time at which the maximum baseline-adjusted hHbi value occurred during exercise was identified. These per-subject peak times were then modeled using linear regression, with the experimental group, age, and gender entered as covariates. Model summaries were generated to assess overall group effects, and estimated marginal means (LS means) for each group were computed and compared pairwise. Group differences in peak timing were visualized using boxplots, and the LS means (with standard errors and confidence intervals) were displayed as bar graphs. LS means (or least-squares means) are the mod-el-predicted average responses for each factor level, adjusted for the other covariates. They represent what the group means would be if all groups had identical distributions of age and gender, allowing fair comparison even in unbalanced designs.

A similar procedure was applied to the absolute peak heights of the baseline-adjusted hHbi values. A Gaussian family generalized linear model was fitted with the same covariates. Estimated marginal means for peak height by group were extracted and visualized with error bars to illustrate adjusted differences in maximum response magnitude.

To examine the full-time course of hHbi, the normality of residuals was first assessed at each time point using Shapiro–Wilk tests.

To analyze how hHbi, cHbi, and O_2_Hbi evolved over time and to compare patterns between the two groups, we utilized a generalized additive model (GAM). Unlike standard linear models, GAMs provide the flexibility to model complex, non-linear relationships between predictors and the outcome, making them highly effective for uncovering subtle or irregular trends over time.

We focused on the change from the baseline as our outcome measure, fitting separate smooth functions for each group to track their progress throughout the observation period. This strategy enabled us to identify and contrast the distinctive patterns of change exhibited by each group. Each model included a smooth function of time stratified by group, along with group, age (or standardized age), and gender as parametric terms, and was estimated under a Gaussian family with restricted maximum likelihood. Model diagnostics and summaries were inspected, and the fitted smooths were plotted to illustrate group-specific trajectories.

For a clearer interpretation, we produced graphical representations of each group’s smoothed outcome trajectory, complete with 95% confidence intervals to reflect the uncertainty around the estimates. Additionally, we calculated the pointwise difference between the groups’ smoothed curves. Regions where the interval did not include zero were flagged (marked these intervals in red on the plot) as indicating statistically significant divergence between groups. The difference curve, overlaid with a shaded ribbon indicating significant intervals, provided a clear depiction of the times at which the groups’ hHbi responses departed significantly from one another.

To examine the associations between two sets of variables, a canonical correlation analysis (CCA) was conducted. The first block, labeled as self-reported fatigue variables (Canadian Criteria for ME/CFS yes/no, increased fatigue self-assessment yes/no, Bell score, Chalder fatigue scale, FACIT fatigue scale), and the second block, labeled as measurement variables (including for each variable (rSO_2_, cHbi, O_2_Hbi and hHbi) the positive and negative areas under the curve, maximum and minimum measurement, the time of these respective measurements and the overall range), were used for the analysis. All variables were first checked for proper formatting and converted to numeric if necessary. Observations with missing values in either block were removed via complete-case analysis. Next, both blocks were standardized to ensure comparability by centering (mean = 0) and scaling (standard deviation = 1). To visualize the results of the canonical correlation analysis, we plotted the subject scores and variable loadings for each block in a common 2D space. First, we determined shared axis limits by combining the CCA1/CCA2 scores and scaled loadings from both the physiological-measurement and self-report blocks. Finally, we extracted the first canonical variate’s loadings for each block and displayed them as horizontal bar plots, ordering the variables by loading magnitude.

All B_delta_cHbi time series were first plotted to display individual trajectories and overlaid with the group mean curve accompanied by a pointwise 95% confidence band; the median trajectory was also inspected for comparison. The overall time window (0–238 s) was then divided into three a priori segments based on the direction change in the mean curve pattern (0–100 s, 100–150 s, and 150–238 s), and for each subject, a separate linear regression of B_delta_cHbi on time was fitted within each segment as well as across the full curve. These per-subject slopes—both stage-wise and full curve—were compared between control and PCS using Welch’s two-sample *t*-tests. All analyses and plots were produced in RStudio (version 2023.09.1 Build 494, ©2009–2023 Posit Software, PBC) using stats and base graphics functions. The following packages were applied: for data rearrangement and extraction: Tidyverse package [30], particularly dplyr; for visualization of graphs: ggplot2 [30] and patchwork [31]; mcgv [32] was used for GAM and GLM modeling; for LS means calculation and comparison of groups after GAM and GLM modeling: emmeans package [33]; and for CCA, vegan [34] and ggvegan [35] packages were used.

## 3. Results

### 3.1. Demographics and Self-Reported Fatigue

The mean age of the PCS group was 44.26 ± 12.55, and 55% were female. The mean age of the control group was 42.64 ± 16.93, with 54% female. Of the 100 patients with PCS, 82% reported suffering from subjective fatigue symptoms on binary screening, and 83% met the Canadian Criteria for ME/CFS. Time since initial positive COVID-19 -PCR averaged 964 ± 275 days in the PCS cohort. Three self-report questionnaires quantifying CF and its impact on daily life were given to all patients, whether they fulfilled the above questions or not. The Bell score, which categorizes activity and activity-associated symptom severity, is scaled from 100 for those fully active without fatigue to 0 for those bedbound by severe symptoms and requiring assistance in all tasks of daily life. Our PCS cohort scored a median of 30 (95% CI 30–40), categorized as suffering from moderate to severe symptoms most of the time but capable of doing about 2 h of activity a day, including tasks that require leaving the house several times a week. The controls had a median Bell score of 100 (95% CI 100–100). FACIT fatigue score is also counter-proportional to fatigue severity, with a high score of 52 for high activity and no subjective tiredness. The mean PCS FACIT score was 20.38 (SD 10.1), while the mean score of the controls was 47.84 (SD 5.05). The Chalder fatigue scale scores are proportional to fatigue severity, with a high score of 11. PCS scored a mean score of 9.964 (SD 2.038), while controls scored a mean of 0.618 (SD 1.670). Demographics and the contrast of fatigue scores are summarized in Table 1.

### 3.2. Oxygenation Changes

**Figure 1:**
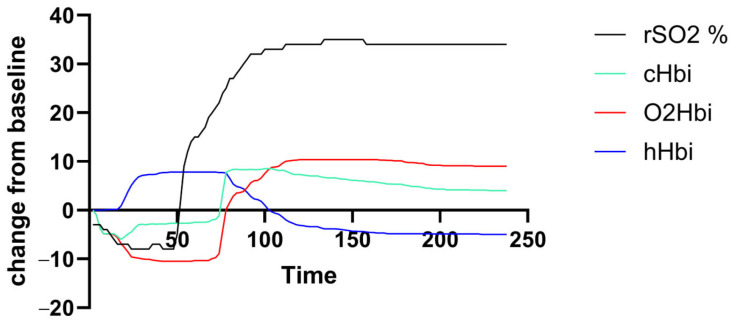
Exemplary output curve of an individual with PCS. There was unfavorable high individual variance in amplitude and curve morphology.

Muscle oxygenation (rSO_2_, cHBi, O_2_Hbi, and hHbi) traces were generated for each participant over the exercise-plus-recovery period. Figure 1 shows a representative deoxygenation–reoxygenation curve from a PCS subject, but there was a high variance in curve amplitude and shape across both groups.

**Figure 2:**
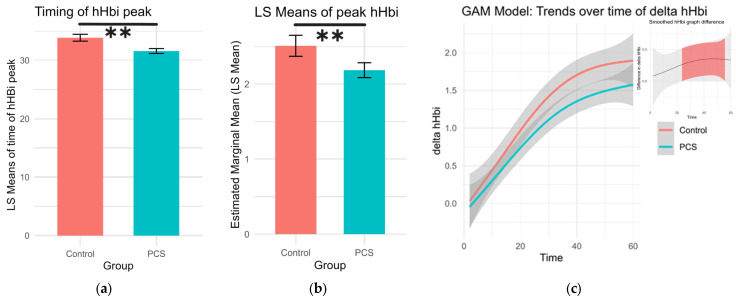
Changes in deoxygenation during exercise. (**a**) The timing of the peak in hHbi_ex_ during the period of exercise. Patients with PCS had significantly earlier peaks (LS means 31.6 s ± 0.416. df 4496, Cl 30.8 to 32.4) compared to controls (LS means 33.9 s ± 0.588 df 4496, Cl 32.7 to 35.0), *p* = 0.0015 (**). (**b**) Peak deoxygenation values during exercise were significantly lower in PCS patients (LS means 2.19 ± 0.0506, df 4496, Cl 2.09 to 2.28), than in controls (LS means 2.51 ± 0.0714, 4496, Cl 2.37 to 2.65), *p* = 0.0002 (**). (**c**) Generalized additive model (GAM) illustrating the trends of change in hHbi during exercise per group. In the top left shows the difference in value as pairwise comparisons of these GAM trend values per time point. The shaded confidence intervals are colored red, where pairwise comparisons yielded a significant difference.

The timing and height of the deoxygenation peak during exercise (hHbi_ex_), which can indicate oxygen extraction efficiency, were analyzed. The highest point of each deoxygenation curve was found along with its respective time point per subject. A Gaussian family generalized linear model was used to calculate differences while age and sex were set as covariates, and the least square (LS) means for sex and group were calculated. PCS patients had earlier but lower hHbi peaks during exercise (*p* = 0.0015, *p* = 0.0002, respectively; Table 2, Figure 2).

To account for high variability in the data and the absence of standard normalization references such as maximal effort or incremental exercise, we applied a series of generalized additive models (GAMs) to examine time-based trends across groups (control vs. PCS).

**Figure 3:**
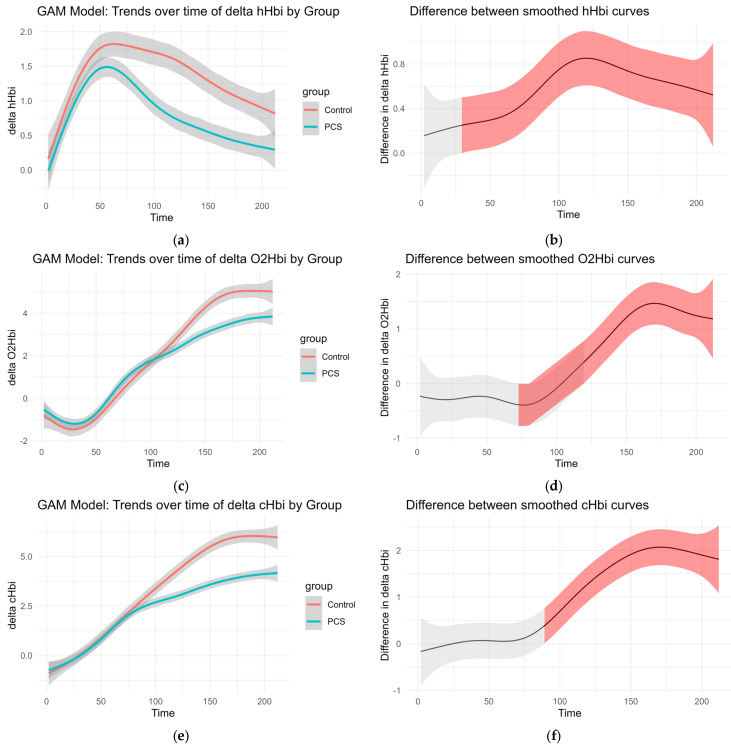

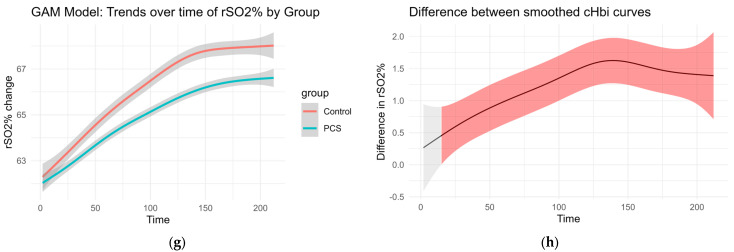
GAM of the overall curves of hHbi, O_2_Hbi, cHbi, and rSO_2_, respectively: (**a**,**c**,**e**,**g**) Change from the baseline plotted over time for each group. (**b**,**d**,**f**,**h**) Graphed difference between the two curves. The shaded area shows the 95% confidence interval and is colored red where there is a significant difference.

Smoothed curves were fitted separately for each group to visualize changes over time in delta O_2_Hb, delta cHb, delta HHb, and regional oxygen saturation (rSO_2_%). These trends are presented in Figure 3. The accompanying difference plots show the estimated difference between group-specific smooths over time, with shaded areas representing 95% confidence intervals. Red shading indicates time intervals where the difference between groups was statistically significant, suggesting divergent physiological responses over time.

This modeling approach reveals group-dependent differences in hemodynamic behavior throughout the observation period, even in the absence of traditional normalization procedures (Figure 3).

**Figure 4:**
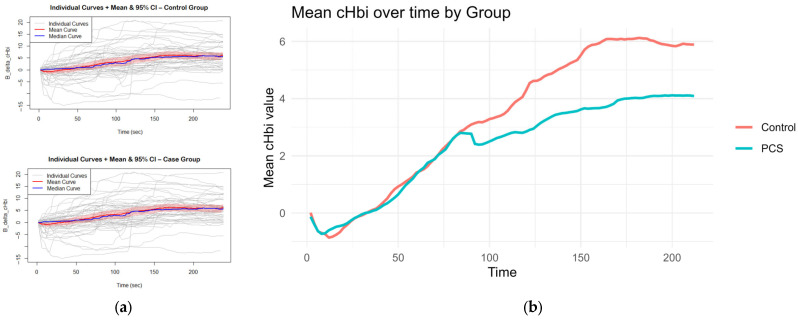
Curves for slope calculation: The mean and median curves of cHbi with 95% CI are shown per group, gray curves show individuals (**a**); Average curves (mean of each group per time point of cHbi) are plotted together, and the overall slope was calculated. Controls exhibited a steeper slope on average *p* = 0.034 (**b**).

CHbi measures the change of total Hb in the investigated tissue. As Mb is localized in the muscle, this is a heavy predictor for hematocrit. Although there is evidence that hematocrit can change somewhat independently of blood flow during exercise, large positive changes in cHbi suggest capillary recruitment and blood flow changes. Therefore, an independent-samples *t*-test was conducted to compare the overall cHbi-time slopes of patients with PCS and controls (Figure 4). There was a statistically significant difference between both two groups (*p* = 0.034, [95% CI: –0.0225, –0.00087]). On average, the control group exhibited a steeper full curve slope (M = 0.03324) than the PCS group (M = 0.02153), indicating that, across the entire time series, hematocrit (as reflected by B_delta_cHbi) increased more rapidly in controls than in PCS. This suggests that the condition defining the PCS group is associated with a measurable slowing of the overall response profile and may hint at perfusion differences.

### 3.3. Multivariate Association Between Self-Reported Fatigue Scores and Masimo-Derived Oxygenation Metrics

**Figure 5:**
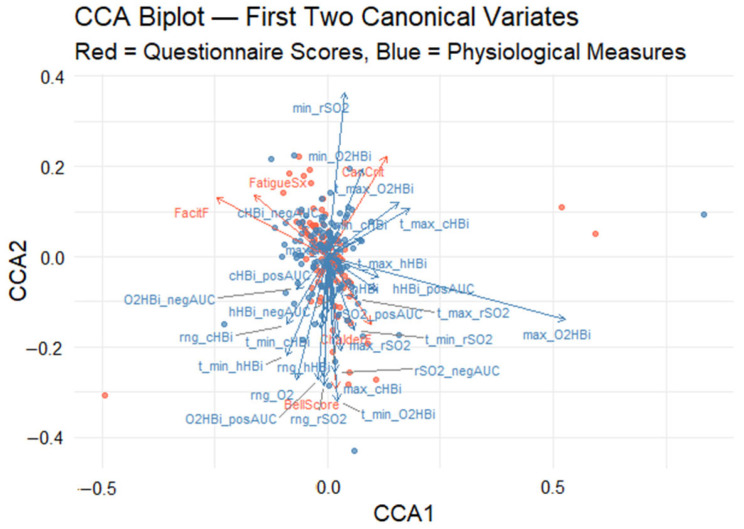
Canonical correlation analysis (CCA) biplot visualizes both (1) the participant scores in the space of the first two canonical variates (CCA1 and CCA2) and (2) the variable loadings (arrows) for each measure. Red points and arrows correspond to the self-reported fatigue scales; blue points and arrows correspond to the oxygenation metrics. Red points show each participant’s CCA1–CCA2 coordinates derived from the five fatigue scale variables, while blue points show the same participants’ coordinates derived from the set of physiological measurements. Red arrows depict how each self-report scale “loads” onto the two canonical axes: arrow length reflects the strength of that variable’s contribution, and arrow direction indicates whether it aligns with CCA1, CCA2, or both. Blue arrows similarly illustrate the loadings of the oxygen measurement variables within the same canonical space; arrows pointing in similar directions are positively correlated, whereas those pointing in roughly opposite directions are negatively correlated.

Canonical correlation analysis (CCA) was used to uncover the multivariate relationship between five self-report fatigue scales (Canadian Criteria, fatigue symptoms, Bell score, Chalder fatigue, FACIT fatigue) and 28 Masimo-derived oxygenation metrics. The analysis revealed a significant association between the two variable datasets, with the first pair of canonical variates yielding a canonical correlation of *rc* = 0.71, as determined by permutation testing (*p* < 0.001). This result indicates a strong latent relationship between subjective fatigue assessments and objective physiological responses. To enhance interpretability, a CCA biplot was generated to visualize the first two canonical dimensions (Figure 5). In this graph, the x-axis and y-axis represent the first and second canonical variates, respectively. Arrows denote the canonical loadings, which correspond to the correlations between the original variables and their associated canonical variates. These are plotted in a shared two-dimensional space to facilitate joint interpretation. Red vectors represent questionnaire-based fatigue scores, while blue vectors represent physiological measures. The distance and direction of each vector from the origin indicate the magnitude and direction of its contribution to the canonical dimensions.

#### 3.3.1. First Canonical Dimension

The first canonical dimension (horizontal axis) biplot explains the largest proportion of the shared variance. On this axis, max_O_2_HBi (maximum oxygenated Hb), cHBi_posAUC (positive deoxygenated Hb in area under the curve), and CanCrit (Canadian Criteria) have the highest positive loadings, suggesting that individuals with the strong endorsement of Canadian diagnostic fatigue criteria tend to show elevated muscle oxygenation responses. In contrast, negative loadings on this axis, particularly from FatigueSx (self-reported fatigue symptom) and FacitF (FACIT fatigue), imply that individuals reporting higher levels of general fatigue tend to exhibit attenuated oxygenated Hb responses.

#### 3.3.2. Second Canonical Dimension

The second canonical dimension (vertical axis) captures a distinct orthogonal pattern of variation, which appears to reflect temporal and depth-related characteristics of desaturation. It is driven positively by min_rSO_2_ (minimum tissue oxygen saturation) and t_max_cHBi (time to the highest total Hb change from the baseline) and negatively influenced by ChalderF score (Chalder Fatigue). The second dimension differentiates individuals based on the dynamics of their oxygenation profiles: those with deeper desaturation versus those whose fatigue is more dominantly expressed using the Chalder scale.

#### 3.3.3. Canonical Correlation Analysis Biplot

The biplot quadrants (Figure 5) offer additional insight into the variation across participants. Individuals located in the upper-right quadrant are characterized by high Canadian Criteria scores and prominent oxygenated hemoglobin excursions, suggesting a physiology-linked fatigue phenotype. Conversely, those in the lower-left quadrant report greater general fatigue severity (as indicated by FatigueSx and FacitF) alongside blunted oxygenation responses, revealing two distinct fatigue physiology phenotypes.

**Figure 6:**
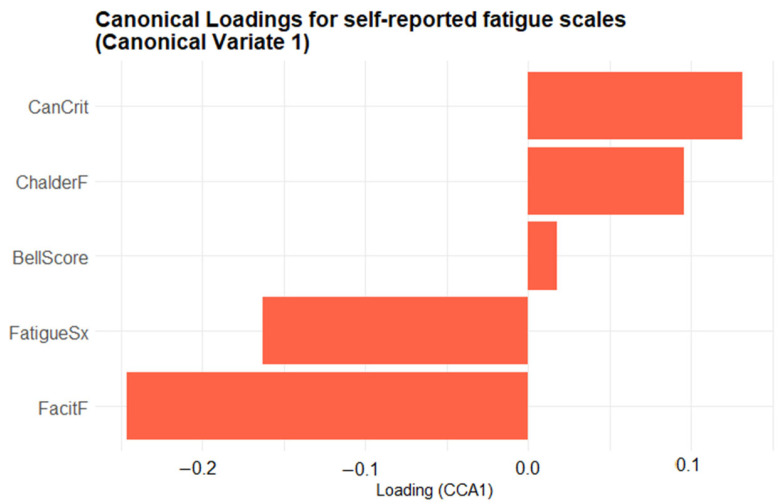
Bar plot of the first-variate canonical loadings for the five self-reported fatigue scales. Each bar shows the Pearson correlation between a questionnaire scale and Canonical Variate 1: its length indicates the strength of that scale’s contribution, and its direction (rightwards = positive, leftwards = negative) indicates whether the scale increases or decreases as the canonical score rises.

Although the biplot enables an intuitive visualization of the joint structure between fatigue and physiological domains, the relevance of each variable is inferred from the relative magnitude and direction of its canonical loading. No explicit threshold was applied; instead, variables represented by vectors farther from the origin are interpreted as exerting greater influence on the corresponding canonical dimension. To complement the biplot, two bar plots were created to provide a clearer depiction of the variable contributions. Figure 6 presents the canonical loadings of the five self-reported fatigue scales on Canonical Variate 1 (CV1), ordered from most negative to most positive, providing a clearer view of each scale’s contribution to the latent dimension. Scales with positive loadings (CanCrit, ChalderF, BellScore) increase as CV1 rises, whereas those with negative loadings (FatigueSx, FacitF) decrease. The length of each bar denotes the magnitude of the Pearson correlation between that scale and CV1, highlighting which questionnaires contribute most strongly to the latent fatigue dimension. The Canadian Criteria scale (CanCrit) loads most strongly in the positive direction, closely followed by the Chalder fatigue scale (ChalderF), indicating that higher scores on these instruments are associated with increases along the primary multivariate axis. The traditional Bell score sits near zero, contributing only marginally, whereas the fatigue symptoms index (FatigueSx) and particularly the FACIT fatigue scale (FacitF) load negatively, signifying that greater self-reported fatigue on those measures corresponds to decreases in the canonical dimension.

**Figure 7:**
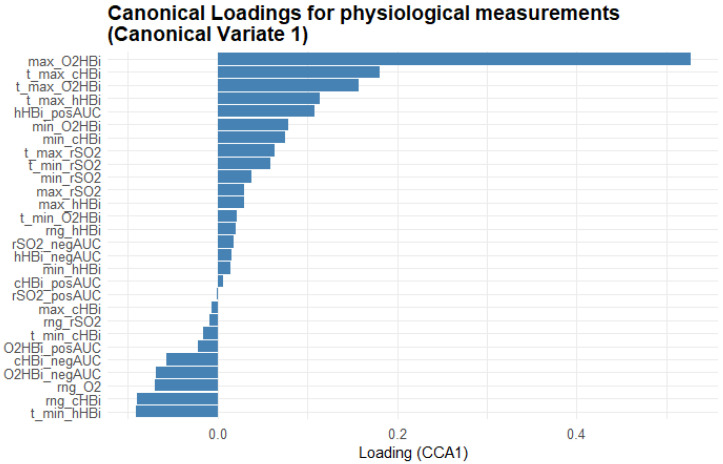
Bar plot of the first-variate canonical loadings for the Masimo physiological measurements. Each bar represents the Pearson correlation between a measurement variable and Canonical Variate 1: its length indicates how strongly that metric contributes to the shared fatigue–physiology dimension and its direction (bars extending right = positive loadings; bars extending left = negative loadings) shows whether the variable rises or falls as the canonical score increases. The variable names are explained in the abbreviations table.

Figure 7 provides a complementary view, showing the Masimo physiological measurements’ loadings on CV1, revealing which oxygenation metrics most closely track the fatigue scales. Maximum oxygenated Hb (max_O_2_HBi) shows the highest positive loading, with its time-to-peak (t_max_O_2_HBi) and the positive area under the curve for total Hb (cHBi_posAUC) also loading strongly positive. In contrast, variables such as minimum deoxygenated Hb (min_hHBi), its time to minimum (t_min_hHBi), and the range of hHBi (rng_hHBi) load negatively, indicating that deeper or earlier drops in Hb saturation oppose the multivariate pattern defined by the fatigue scales.

## 4. Discussion

PCS is a post-viral disorder, summarizing several subgroups. One of them is characterized by its clinical major symptom, PEM [3]. It is hypothesized that a restricted microcirculation, accompanied by ischemia-reperfusion, might induce or maintain PEM. Recent data yielded evidence that a subgroup of patients with PCS showed an impaired capillary microcirculation mapped on the retina by OCT-angiography [13,16]. It assumed that a special autoimmune phenomenon characterized by functional autoantibodies targeting G-protein coupled receptors (GPCR-fAAb), accompanied by ischemia, triggers clinical PCS symptoms. This hypothesis was substantiated first by a successful healing attempt with Rovunaptabin (BC007) [17] and, afterward, a clinical phase IIa study [18], showing an improvement of fatigue after neutralization of GPCR-fAAb. As PCS diagnosis lacks objective biomarkers to date, the aim is to establish novel biomarkers for the visualization of oxygenation deficiencies. Thus, the aim was to investigate the (de)oxygenation of patients with PCS compared with controls during and after light exercise.

Analysis of our extensive cohort revealed that even light exercise elicits differences in oxygenation responses and local hematocrit in patients with PCS compared to controls. These differences appeared to become more pronounced during recovery after short exercise and resulted in a lower rSO_2_ trend by the end of observation in PCS. Some of these differences, most strongly the peak of O_2_Hbi, were associated with self-reported fatigue questionnaires, further supporting the link between fatigue and oxygenation deficiencies and mitochondrial and endothelial dysfunction.

NIRS is clinically used to assess oxygenation in patients in intensive care or during cardiac surgeries, as it non-invasively and continuously measures the oxygenation state of hemoglobin within tissues and the regional oxygen saturation [36,37]. In devices that support two detectors per sensor, deep tissues can be selectively monitored according to the modified Lambert–Beer Law. Thus, the depth of investigation is tied to the detector spacing, which can lead to difficulty in assessing patients with very low or very high BMI [19]. Correct sensor placement and secure adherence are necessary for stable measurements. Furthermore, the absorption spectra of Hb and Mb overlap, so the variables reflect the oxygenation state of both. In healthy individuals, however, Mb oxygenation is assumed to remain at the same levels during exercise, so changes from the baseline oxygenation are expected to reflect only hemoglobin oxygenation [19]. The most studied variable in literature is rSO_2_, as only newer generation devices can differentiate between changes in O_2_Hb, hHb, and cHb [38].

Previous investigations used incremental or maximal exercise [29,39], fractions of the maximum voluntary contractions [40], or vascular occlusion [7] to measure muscle oxygen consumption. Implementation of light exercise avoids triggering PEM. As the clinical need for diagnosis and follow-up of patients with PCS and PEM is high and PEM worsens abnormal muscle findings [4], implementation of a protocol, which even wheelchair-bound PCS patients were able to follow, would be recommended.

In healthy individuals, during exercise, rSO_2_ decreases as an acute response to heavy exercise. At high aerobic exercise rates, microvascular blood flow plateaus and dissociates from arterial blood flow; in addition, the muscle oxygen extraction exponentially decreases after sudden cessation of the exercise [20]. At rest, fractional O2 extraction is said to be ~25% and increases to ~75% during maximal exercise [20].

HHb increases sharply at the start of exercise and can be used as a semiquantitative estimation of the difference between oxygen concentration in arteries and veins [20]. This overshoot is increased in chronic heart failure and peripheral artery disease, leading to impaired O2 diffusion due to decreased microvascular oxygen partial pressure [41]. In myopathies like mitochondrial myopathy or McArdle disease, this hHb overshoot and hHb max are reduced, suggesting excess blood flow for the same work rate, key pathological mechanisms of these diseases [42]. In the present cohort of patients with PCS and PEM, a decreased hHbi max was observed, suggesting impaired muscle oxygen extraction (Figure 2). It could be hypothesized that this might be due to endothelial or mitochondrial dysfunction.

Muscle oxygen extraction follows an exponential decrease during recovery after exercise. This reoxygenation has been postulated to be a tool for the evaluation of functional oxidative performance, as it correlates well with creatine phosphate recovery, a molecule classically used in physiology for the evaluation of skeletal muscle oxidative metabolism [43]. This exponential increase of O_2_Hbi is likely reflected in our data as a trend. In controls, this steepness was greater in the O_2_Hbi curve, and the amplitude at the end of observation was higher, potentially suggesting impaired oxidative metabolism during recovery within the PCS cohort. Slowed reoxygenation kinetics but no oxygenation differences during exercise have also been observed in Friedreich’s ataxia [44] and Fibromyalgia [45]. Friedreich’s ataxia is a genetic disease characterized by mitochondrial damage with early symptoms of fatigue, spasticity, and muscle weakness. Fibromyalgia is characterized by increased muscle pain, fatigue, decreased exercise tolerance, loss of concentration, and small fiber neuropathy. These findings were also observed in our patients with PCS, further suggesting a link between impaired reoxygenation and PEM [4].

The multifaceted nature of self-reported fatigue is challenging as there is a lack of objective diagnostic tools for visualization and quantification. Fatigue is reported to be involved in diverse disorders (e.g., cancer) with known and partially unknown pathology. PEM, as one feature of fatigue, is typically characterized by delayed fatigue and characterizes a subgroup of patients with PCS and ME/CFS.

In addition, mental fatigue is one symptom of ‘brain fog’, which is associated with immune-mediated disorders (e.g., systemic lupus erythematosus [46]). Considering this aspect, functional NIRS (fNIRS) could potentially be a viable option to measure mental fatigue in patients with PCS. The use of fNIRS to measure fatigue has been reviewed by Yan et al. [47]. Previously, most studies measured changes from the baseline during fatiguing tasks. As PCS patients complain of chronic brain fog and are likely to have perfusion alterations, it is unclear if simply measuring changes from the baseline would yield results. Currently, most fNIRS devices are custom-built and incorporate EEG. EEG and the use of a frequency domain NIRS emitter, capable of detecting the absolute tissue concentration of hemoglobin, would help find a potential baseline and differences as well.

A previous study investigating muscle tissue oxygenation of the vastus lateralis in patients with PCS was previously performed, but it did not show significance compared to controls [48]. Yet, the cohort size was lower (36 PCS, 11 controls), and measurements were conducted every 30 s during standing and walking (6 min walking test) and after exercise [48].

In addition, previous data offer that quantification of tissue oxygenation might be best reflected in the slopes of the oxygenation curves after vascular occlusion [7].

Interindividual variability is known to be high in NIRS measurements, largely due to two factors: the limitation in applying the modified Lambert–Beer law is that each individual’s optical scatter value is not known and is likely subject to alteration with changes in blood flow. Further on, the relative contribution of arterial, venous, and capillary hemoglobin to the measurement depends on the placement of the sensor on the individual and reactive hyperemia during exercise [19].

In response to exercise perfusion, the total Hb concentration of the affected area is increased, which is associated with an increase in total Hb (i.e., cHbi). Local hematocrit can even change independently of blood flow during exercise, which is a phenomenon that is not fully elucidated. As the data of the present study showed an increase of cHbi in controls compared to patients with PCS, this could potentially reflect a slower capillary recruitment. It can be hypothesized that this might be a consequence of microclot load, altered physical blood cell characteristics, or endothelial dysfunction. Consequently, the impaired oxygen uptake of the muscles might be mediated by defects in the cellular respiration of skeletal muscle, reduced diffusion across the vascular wall, or a combination of these factors.

In summary, the data of the present study show that NIRS might be a potentially viable tool to assess vascular dysregulation after exertion in patients with PCS. Further clinical studies are necessary, investigating different light exercise durations and their long-term recovery effects.

## 5. Conclusions

PCS is a challenging disorder, as the diagnosis lacks objective biomarkers up to date. As the data of the present study offered that the subgroup of patients with PCS and PEM showed impaired deoxygenation after light exercise, these data support the hypothesis of ischemia as a pathogenetic factor in PEM. Visualization and quantification of this restricted deoxygenation might offer a diagnostic tool in this subgroup of patients with PCS, showing PEM as a major clinical symptom. In addition, this test setup might be useful for bedbound patients.

## Figures and Tables

**Table 1:** Demographic data of participants and questionnaire scores.

Variable	PCS	Control
Age	44.28 (19–78; SD 12.55)	42.64 (22–68; SD 16.93)
Sex	55% female	54% female
Time from positive PCR test to investigation (days)	909 (126–1565)	N/A ^1^
Canadian Criteria	83% fulfillment	0% fulfillment
Median Bell score	30 (95% CI 30–40)	100 (95% CI 100)
FACIT fatigue score	20.38 (SD 10.1)	47.84 (SD 5.05)
Chalder fatigue scale	9.964 (SD 2.038)	0.618 (SD 1.670)

^1^ Control group constituted of people who either never had COVID-19 or who had fully recovered from COVID-19.

**Table 2:** Estimated coefficients, standard errors, t-values, and *p*-values from the GLM models of Time point and peak of deoxygenated hemo-and myoglobin baseline change (hHbi) during exercise, as a function of the intercept, PCS group, female sex, and age.

Variable				
Time of Peak hHbi_ex_	Estimate	Std. Error	T Value	Pr(>|t|)
Intercept	27.67810	1.22680	22.561	<2 × 10^−16^
Group PCS	2.27568	0.71980	−3.162	0.00158
Age	0.12032	0.68123	2.695	0.00706
Sex Female	1.83608	0.68123	2.695	0.00706
**Peak hHbi_ex_ value**				
Intercept	1.83154	0.14899	12.293	<2 × 10^−16^
Group PCS	−0.32504	0.08742	−3.718	0.000203
Age	0.01890	0.00293	6.450	1.23 × 10^−10^
Sex Female	−0.29506	0.08273	−3.566	0.000366

## Data Availability

The datasets generated and/or analyzed during the current study are available from the corresponding author, B.H., on reasonable request.

## Abbreviations

The following abbreviations are used in this manuscript:

PCS	Post-COVID-Syndrome
PEM	Post-exertional malaise
NIRS	Near-infrared regional spectroscopy
LC	Long COVID
CF	Chronic fatigue
GPCR-fAAb	Functional autoantibodies targeting G-protein coupled receptors
Hb	Hemoglobin
Mb	Myoglobin
PCR	Polymerase chain reaction
ME/CFS	Myalgic Encephalomyelitis/Chronic Fatigue Syndrome
LFT	Lung function test
LVEF	Left ventricular ejection fraction
Delta O_2_Hbi	Change in oxygenated hemoglobin/myoglobin
Delta hHbi	Change in deoxygenated hemoglobin/myoglobin
Delta cHbi	Total change in hemoglobin/myoglobin
rSO_2_	Regional oxygen saturation
GAM	Generalized additive model
CCA	Canonical Correlation Analysis
AUC	Area under the curves
rSO_2__posAUC	Positive area under the curve of regional oxygen saturation
rSO_2__negAUC	Negative area under the curve of regional oxygen saturation
t_min_rSO_2_	Time to minimum rSO_2_ during observation
t_max_rSO_2_	Time to maximum rSO_2_ during observation
max_rSO_2_	Maximum rSO_2_ value during observation
min_rSO_2_	Minimum rSO_2_ value during observation
Rng_rSO_2_	Range of rSO_2_ values across all time points
cHbi_posAUC	Positive area under the curve of total change in cHbi
cHbi_negAUC	Negative area under the curve of total change in cHbi
t_min_cHbi	Time to minimum cHbi during observation
t_max_cHbi	Time to maximum cHbi during observation
max_cHbi	Maximum cHbi value during observation
min_cHbi	Minimum cHbi value during observation
rng_cHbi	Range of cHbi values across all time points
O_2_Hbi_posAUC	Positive area under the curve of total change in O_2_Hbi
O_2_Hbi_negAUC	Negative area under the curve of total change in O_2_Hbi
t_min_O_2_Hbi	Time to minimum O_2_Hbi during observation
t_max_O_2_Hbi	Time to maximum O_2_Hbi during observation
max_O_2_Hbi	Maximum O_2_Hbi value during observation
min_O_2_Hbi	Minimum O_2_Hbi value during observation
rng_O_2_Hbi	Range of O_2_Hbi values across all time points
hHbi_posAUC	Positive area under the curve of total change in hHbi
hHbi_negAUC	Negative area under the curve of total change in hHbi
t_min_hHbi	Time to minimum hHbi during observation
t_max_hHbi	Time to maximum hHbi during observation
max_hHbi	Maximum hHbi value during observation
min_hHbi	Minimum hHbi value during observation
rng_hHbi	Range of hHbi values across all time points
hHbi_ex_	Deoxygenated hemo/myoglobin during exercise
LS means	Least square means
CV1	Canonical variate 1
CanCrit	Canadian Criteria for ME/CFS
FacitF	FACIT fatigue score
FatigueSx	Self-reported fatigue symptom
ChalderF	Chalder fatigue scale
fNIRS	Functional near-infrared regional spectroscopy
